# Current Status, Challenges, and Perspectives in the Conservation of Native Honeybees and Beekeeping in Cambodia

**DOI:** 10.3390/insects16010039

**Published:** 2025-01-03

**Authors:** Eric Guerin, Chhouk Chheang, Chainarong Sinpoo, Korrawat Attasopa, Nuttapol Noirungsee, Huoqing Zheng, Tial C. Ling, Patcharin Phokasem, Terd Disayathanoowat

**Affiliations:** 1 Asian Native Honey Bee Conservation and Sustainable Beekeeping, Bantey Chas Village, Slor Kram Commune, Siem Reap 171201, Krong Siem Reap, Cambodia; eric.guerin68@gmail.com; 2 Department of Agronomy, Faculty of Agriculture and Food Processing, National Meanchey University, Sisophon 010807, Banteay Meanchey, Cambodia; chheang.chhouk.mcu@moeys.gov.kh; 3 Office of Research Administration, Chiang Mai University, Chiang Mai 50200, Thailand; chainarong.s@cmu.ac.th; 4 Department of Biology, Faculty of Science, Chiang Mai University, Chiang Mai 50200, Thailand; nuttapol.n@cmu.ac.th; 5 Research Center of Deep Technology in Beekeeping and Bee Products for Sustainable Development Goals (SMART BEE SDGs), Chiang Mai University, Chiang Mai 50200, Thailand; korrawat.a@cmu.ac.th; 6 Department of Entomology and Plant Pathology, Faculty of Agriculture, Chiang Mai University, Chiang Mai 50200, Thailand; 7 College of Animal Sciences, Zhejiang University, Hangzhou 310027, China; hqzheng@zju.edu.cn; 8 Department of Environmental and Plant Biology, Ohio University, Athens, OH 45701-2979, USA; tcling@ohio.edu

**Keywords:** bee conservation, beekeeping, honey hunting, cambodia, challenges, policies perspectives

## Abstract

The four honeybee species native to Cambodia—*Apis dorsata*, *Apis cerana*, *Apis florea*, *and Apis andreniformis*—play a vital role in ecosystem health and agricultural productivity through their pollination activities. Beekeeping in Cambodia has primarily developed around the introduced species *Apis mellifera*. However, it remains underdeveloped compared to neighboring countries, with wild honey collection continuing to play a significant role. Although native bees are not at immediate risk of extinction, their crucial ecological and socio-economic roles, coupled with the threats to their populations, necessitate the urgent implementation of conservation policies. The beekeeping sector in Cambodia faces numerous challenges that hinder its development. These include a lack of documentation and official data, limited access to productive apiaries, inappropriate pesticide practices, insufficient knowledge about bee health, diseases, and parasites, inadequate organization and representation of beekeepers, the high cost of Cambodian honey, and a lack of consumer awareness and trust in local bee products. This paper provides an overview of the status and distribution of honeybee species, along with the production, value chain, and trade of bee products in Cambodia. It examines the challenges of conserving native honeybees and developing the beekeeping sector while proposing strategies to strengthen bee conservation and support the growth of beekeeping in the country. Key recommendations focus on integrating bee conservation strategies with broader conservation goals, such as reducing deforestation, promoting sustainable agriculture, and regulating the consumption of wild bee brood. Key strategies for the development of beekeeping are documenting and conserving bee floral resources and habitats, reducing the use of bee-toxic pesticides, developing research and training capacities in beekeeping, regulating bee imports, and enhancing quality and authenticity testing facilities. Additionally, building capacity among honey value chain stakeholders in areas such as quality control, authenticity, processing, packaging, and marketing is essential.

## Simple Summary

Cambodia is home to four native species of honeybees (*Apis dorsata*, *A*. *cerana*, *A*. *florea* and *A*. *andreniformis*) alongside the introduced *A*. *mellifera*, all of which play a vital role in pollinating the country’s forests and crops, thereby supporting biodiversity and agricultural productivity. However, native honeybee populations face mounting challenges, including habitat loss, agriculture intensification, unsustainable honey harvesting, and limited conservation initiatives. Beekeeping in Cambodia remains underdeveloped compared to neighboring countries, with wild honey collection continuing to play a significant role due to its cultural significance and the perceived medical value of wild honey. This review examines the status and distribution of honeybee species, assesses the threats to their populations, and discusses strategies for their conservation. It also evaluates the status of honey production value chain and trade, along with the challenges faced by the beekeeping sector, while proposing strategies for its development in Cambodia.

## 1. Introduction

The global loss of honeybee (*Apis* species) colonies, driven by climate change, habitat loss, anthropogenic disturbances, and the spread of diseases and pests, poses a significant threat to biodiversity and agriculture worldwide [1,2]. Honeybees are pivotal for the pollination of a wide array of crops and wild plants, underpinning ecosystem services essential for food security and ecological health. In Asia, the diversity and distribution of honeybees reflect a rich tapestry of native and introduced species [3], each playing a unique role in their respective ecosystems and facing distinct conservation challenges. In Cambodia, the varied geography, ranging from lush forests and fertile plains to diverse ecosystems, offers an ideal habitat for various bee and other animal species [4]. Notably, the country is home to five species of honeybees, whose presence spans multiple provinces and is crucial for the pollination that enhances the biodiversity and productivity of the country’s flora [4,5,6]. These species include the native *Apis dorsata* (the Asian giant honeybee), *Apis cerana* (the Eastern honeybee), *Apis florea* (the red dwarf honeybee), and *Apis andreniformis* (the black dwarf honeybee), as well as the introduced *Apis mellifera* (the Western honeybee), the last of which has become the integral to modern beekeeping practices [6]. With the modernization of agriculture and the growing environmental challenges, beekeeping in Cambodia faces numerous obstacles that hinder its progress [7]. Traditional practices, such as the harvest of wild honey, which is reversed for its therapeutic and medicinal properties [8], coexist with modern beekeeping, yet often overshadow it due to the cultural and economic value placed on wild honey [6]. This dynamic creates challenges that are exacerbated by the prevalence of bee diseases (e.g., chalkbrood) and parasites (e.g., *Varroa* and *Tropilaelaps* mites), which affect bee health and productivity [9]. Despite the recognized value of honeybee products for their medicinal and therapeutic properties, both locally and internationally, Cambodia’s beekeeping sector remains underdeveloped compared to its neighbors. This lag in development is marked by a lack of comprehensive studies assessing the overall impact of bee health issues within the country and those addressing the root causes of bee decline. This situation highlights the critical need for targeted conservation efforts, which are significantly less advanced than those seen in other regions such as Europe, North America, and neighboring Asian countries like China, Singapore, Thailand, and Vietnam [3]. The four native honeybee species, while not currently at risk of short-term extinction, play crucial socio-economic and ecological roles that are threatened by these ongoing challenges. The urgent need for enhanced conservation policies is thus underscored by the potential loss of these valuable ecological and economic resources. This review assesses the status, challenges, and future directions for conserving native honeybee populations and enhancing honey production in Cambodia. By analyzing existing literature and survey data, we focus on the distribution of native honeybee species, beekeeping practices, and opportunities for sustainable honey production. This paper aims to provide a comprehensive overview of the current conservation efforts for Cambodia’s native honeybees, highlighting key challenges, and proposing actionable recommendations for improving conservation strategies and sustainable production in alignment with socio-economic and environmental sustainability objectives. Significantly, this study also aims to fill the knowledge gaps by comparing the situation in Cambodia with that in other Asian countries, thus situating the local challenges within a broader regional context.

## 2. Current Status, Distribution, and Conservation of Honeybees in Cambodia

**Figure 1:**
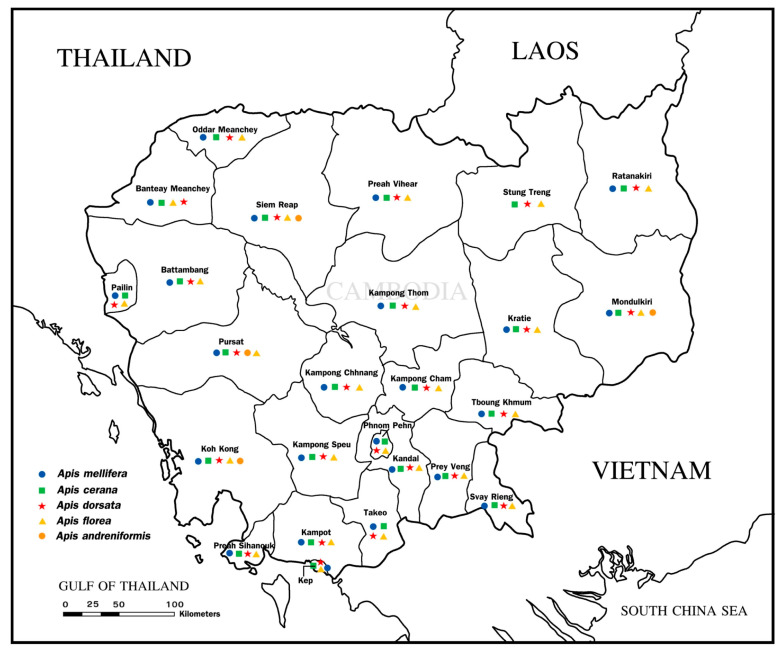
Provinces and the occurrences of the five honeybee species in Cambodia.

In Cambodia, the four native species of honeybees each contribute uniquely to the local ecology and agriculture. Additionally, the introduction of *Apis mellifera* plays a crucial role modernizing the country’s beekeeping practices. The ecological and agricultural significance of these honeybees cannot be understated; they are crucial for the pollination of both wild floral and agricultural crops, which is essential for maintaining biodiversity and enhancing food security in the region. Their pollination services support not only the ecological health of natural habitats but also the productivity of agricultural systems, which as the backbone of Cambodia’s rural economy. Given their vital roles, understanding the distribution and conservation status of these honeybees is paramount for developing effective management and conservation strategies. This ensures not only the survival of the bee populations but also the sustainability of agricultural practices and natural ecosystems that depend on them. According to the Global Biodiversity Information Facility (GBIF) [10] and supplemented by numerous studies [5,6,11,12,13,14], these honeybees have distinct provincial distributions, as illustrated in Figure 1. *Apis dorsata*, *Apis florea*, and *Apis cerana* are found throughout the country. *Apis andreniformis*, with a more limited distribution, is observed in four provinces, including Mondulkiri, Siem Reap, Pursat, and Koh Kong. Our recent survey data further indicate the widespread presence of the introduced *A. mellifera* across nearly every region, with colonies documented in 24 provinces: Oddar Meanchey, Siem Reap, Pailin, Battambang, Pursat, Kampong Thom, Kampong Cham, Kampot, Takeo, Phnom Penh, Kandal, Svay Rieng, Prey Veng, Tboung Khun, Mon-dulkiri, Banteay Meanchey, Preah Vihear, Kratie, Kampong Speu, Kampong Chhnang, Koh Kong, Ratanakiri, Kep, and Preah Sihanouk. This broad distribution underscores the adaptability of *A. mellifera* and its significant impact on local beekeeping practices.

### 2.1. Apis dorsata (Asian Giant Honeybee)

**Figure 2:**
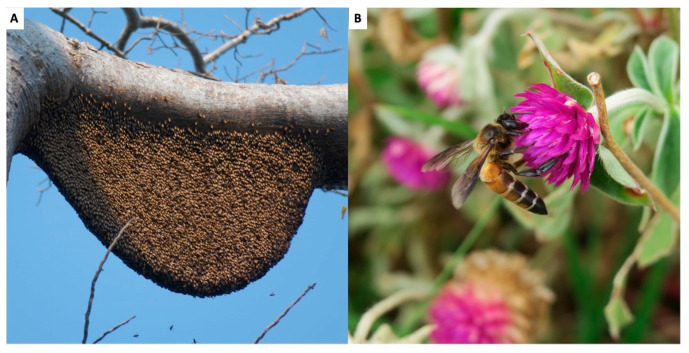
Asian giant honeybee (*Apis dorsata*): (**A**) Nest (**B**) Worker bee. Photo by Eric Guerin.

*Apis dorsata*, known for its large open-air nests, remains a significant species within the Cambodian apicultural landscape, contributing notably to medicinal practices (Figure 2). As previously noted, this species is widely distributed across Cambodia. Despite the widespread presence of *A*. *dorsata* in this country, it is highly sensitive to environmental changes, leading to population decline due to deforestation and intense hunting pressures [6,15,16]. These declines vary significantly across different regions of the country, with some areas (e.g., Tonle Sap Biosphere Reserve) experiencing more severe reductions than others (e.g., Mondulkiri and Koh Kong provinces) where populations remain relatively abundant due to well-preserved forests and sustainable honey harvesting practices [17]. Data on the ecological behavior of *A*. *dorsata* illustrate its vulnerability and the complexity of its life cycle [18]. However, migration patterns of *A*. *dorsata* colonies in Cambodia, which are essential for their survival and reproduction, remain poorly understood. Initial records have suggested that these colonies disperse throughout the country during the dry season and gather in specific habitats like the flooded forests of Tonle Sap Lake and the coastal Melaleuca (*Melaleuca leucadendra*) forests during the rainy season [6,15,17]. However, these preliminary findings require confirmation through additional comprehensive surveys. Particularly in the Tonle Sap Biosphere Reserve, the colonies engage in complex migrations influenced by flower blooms and fluctuating water levels, involving several nesting sites within the reserve and movements between the flooded forest and upland areas [6].

The pressing decline of *A*. *dorsata* populations is highlighted by the decreased frequency and size of colony aggregations, with recent observations of small groups of around 20 colonies contrasting sharply with historical accounts from 1948 that described large aggregations of up to 90 nests [6,17,19]. This reduction has been compounded by many honey hunters reporting a decrease in colony size, likely a result of excessive hunting pressure and the systematic, repetitive destruction of bee nests through unsustainable harvesting practices [6]. These challenges severely impair the colonies’ growth and the size of the swarms they produce.

### 2.2. Apis cerana (Eastern Honeybee)

**Figure 3:**
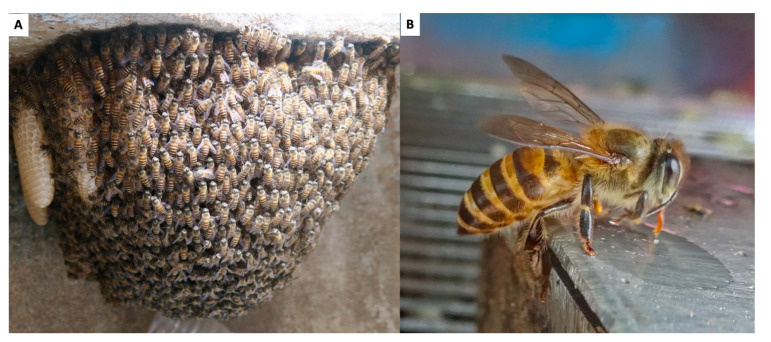
Asian Honeybee (*Apis cerana*): (**A**) Colony (**B**) Worker bee. Photo by Eric Guerin.

*Apis cerana* is widespread in Cambodia, although its populations seem to be less dense compared to those in neighboring countries like Laos, Thailand, and Vietnam [15,20]. This is suggested by the limited success in capturing *A. cerana* colonies in several provinces of Cambodia compared to the large number of colonies successfully captured annually by beekeepers in neighboring countries. It is likely that the relatively low abundance of *A. cerana* in Cambodia is an adaptation to what is perceived as the country’s limited floral resources, although empirical studies are needed to validate this hypothesis. The absence of beekeeping tradition with *A. cerana* within the country could be explained by the perceived floral scarcity [6]. Notably, *A. cerana* is abundant only in the western parts of Cambodia along the Thai border, where more fertile land and a wetter climate support a greater abundance and variety of bee forage plants. Conversely, *A. cerana* was found to be absent from the flooded forest of the Tonle Sap Biosphere Reserve [6], likely due to a lack of suitable cavities above flood level in these forests [6]. *Apis cerana* colonies (Figure 3) periodically relocate their nest [21,22], resulting in a high absconding rate among managed colonies. Although the specific migration routes of *A. cerana* populations in Cambodia are not yet known [6], genetic studies have shown that these populations belong to the Mainland Asia lineage, closely related to those in P.R. China, Laos, Myanmar, Vietnam, and Thailand [14,23,24].

The specific role of *A*. *cerana* in pollinating unique Cambodian crops and native plants highlights its ecological significance. *Apis cerana* can be equally important in pollinating fruit crops such as lychee, mango, longan, sesame, corn, coconut, cashew nuts, and coffee, which are significant to Cambodia’s agricultural economy, although empirical evidence is needed to validate this hypothesis. However, the scarcity of *A*. *cerana* could lead to reduced pollination services, particularly native plants, for pollinators ultimately impacting fruit yields and agricultural economy. This scarcity could also affect the biodiversity of native plants that rely on this species for pollination, potentially leading to reduced seed set and less genetic diversity within plant populations. Conservation efforts should, therefore, prioritize habitat protection and the management of threats such as pesticide use and habitat destruction to maintain the ecological balance and support agricultural productivity.

### 2.3. Apis florea (Red Dwarf Honeybee)

**Figure 4:**
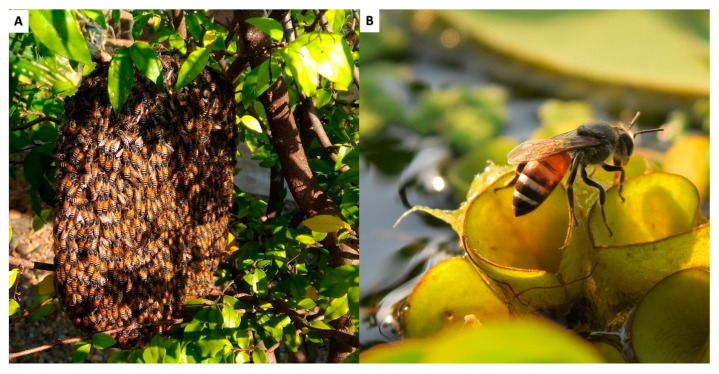
Red dwarf honeybee (*Apis florea*): (**A**) Nest (**B**) Worker bee. Photo by Eric Guerin.

*Apis florea*, the red dwarf honeybee (Figure 4), is commonly found in agricultural areas, which is why it might be vulnerable to pesticide exposure [25]. It has been observed that Cambodian honey hunters, typically targeting *A. dorsata*, shift their focus to *A. florea* when *A. dorsata* colonies become scarce [6]. Thus, with the progressive decline of *A*. *dorsata* populations, there is an increasing risk of *A. florea* experiencing population declines as well [6]. According to morphometric analysis, *A. florea* populations in Cambodia are genetically similar to those in Myanmar, Thailand, and northern Vietnam [26]. To mitigate the impact of pesticides on *A*. *florea* populations, agricultural stakeholders could be encouraged to adopt integrated pest management (IPM) practices that minimize pesticide use. Establishing pesticide-free zones around key *A. florea* habitats, especially in regions where they are most abundant, would help preserve their populations. Community education programs could be implemented to raise awareness among farmers about the critical role of bees in pollination and the dangers of pesticide overuse. Regulatory changes might include stricter controls on pesticide sales and the introduction of buffer zones around protected areas. For instance, the Cambodian Ministry of Agriculture could enforce regulations that restrict the types of pesticides used near known bee habitats to bee-safe alternatives.

### 2.4. Apis andreniformis (Black Dwarf Honeybee)

**Figure 5:**
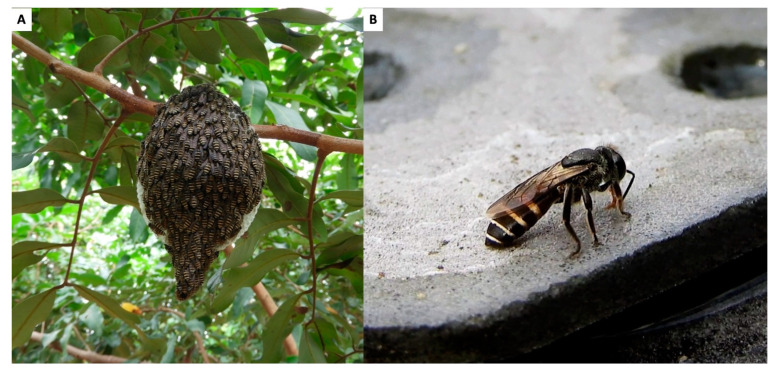
Black Dwarf Honeybee (*Apis andreniformis*): (**A**) Nest (**B**) Worker bee. Photo by Chhouk Chheang.

*Apis andreniformis* is the rarest and most sporadically distributed of Cambodia’s native honeybee species. To date, its presence in the kingdom has been documented in only four provinces: Koh Kong, Mondulkiri, Pursat, and Siem Reap [5] (Figure 5). Although altitudinal migrations of *A. andreniformis* have been documented in Thailand, where they nest at higher altitudes in the rainy season and lower altitude in the dry season [27], such migratory patterns have not yet been observed in Cambodia. Given the limited understanding of *A*. *andreniformis* behavioral patterns and ecological needs, there is an urgent need for targeted research. Studies should focus on their nesting habits, dietary preferences, and role within local ecosystems. Exploring how this species adapts to different environmental conditions across Cambodia, particularly in response to climate variability, is crucial. Additional research into the reproductive biology and genetic diversity of *A*. *andreniformis* will help inform conservation strategies that can address their specific needs and vulnerabilities. Conservation biologists and local universities could collaborate on long-term monitoring projects to track population changes and habitat use patterns, thereby developing effective conservation strategies for this lesser-known species.

### 2.5. Apis mellifera (Western Honeybee)

*Apis mellifera* was introduced to Asia, including Japan, India, and Indonesia, in the late 1800s and has been widely adopted for beekeeping across the continent since the 1980s [9,28,29]. In recent decades, *A. mellifera* has been introduced to Cambodia from Vietnam and Thailand to enhance modern beekeeping practices [6]. It is now widespread throughout the country, with thousands of colonies imported annually, although the exact numbers remain uncertain. The genetic lineage of *A. mellifera* in Cambodia remains unclear.

As mentioned earlier, *A*. *mellifera* is a non-native species and it may compete with native bee species for floral resources, particularly in areas where flower abundance is limited. This competition can potentially lead to reduced food availability for native bees, impacting their survival and reproduction. Moreover, the introduction of *A*. *mellifera* has been linked to increased transmission of pathogens to native bees, with diseases such as the deformed wing virus having been identified in native bee populations following the introduction of *A*. *mellifera*. A recent report documented the presence of DWV types A and B in native honeybee species located near *A. mellifera* colonies, leading to its widespread transmission among *Apis* species [30]. The virulence of DWV isolated from wild honeybees (*A. mellifera*) was found to be lower compared to that in managed honeybees [31]. However, monitoring programs, such as those conducted by the Cambodian Department of Environment, are essential to assess the health of bee populations across regions where *A*. *mellifera* has been introduced. These programs can help identify any negative impacts early and facilitate the development of management strategies to mitigate these effects, such as controlling the movement of commercial *A*. *mellifera* colonies and enhancing biosecurity measures to prevent disease spread.

### 2.6. General Considerations

The current status of honeybees in Cambodia reflects a complex interplay of ecological, economic, and cultural factors influencing their conservation and management. While some species like *A*. *dorsata* and *A*. *florea* are deeply embedded in traditional practices, others like *A*. *mellifera* represent modern agricultural adaptations. The conservation status varies significantly across species, with habitat loss, human activities, and climate change being predominant threats. Moving forward, comprehensive and updated data are crucial to better understand these dynamics and to implement effective conservation strategies. Studies should aim to cover not only population assessments but also the ecological impacts of both native and introduced species on local ecosystems.

## 3. Bee Forage Resources

Cambodia’s floral resources, crucial for sustaining a diverse and healthy bee population, remain poorly documented, underscoring the need for further research and assessment.

## 4. Honey Hunting and Beekeeping in Cambodia

### 4.1. Historical Record of Honey Production in Cambodia

**Figure 6:**
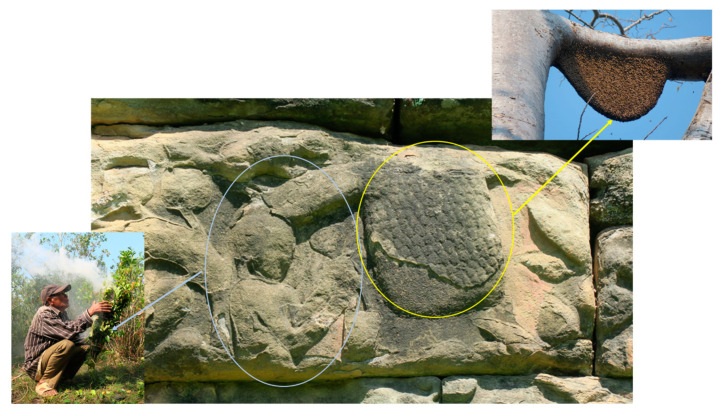
Honey hunting scene carved on the bas-reliefs of the Elephants Terrace at Angkor Thom, Siem Reap. The bas-relief illustrates the traditional practice of honey hunting in Cambodia. The yellow circle highlights an *Apis dorsata* colony, showcasing its characteristic single, large comb hanging from a branch, while the blue circle emphasizes a honey hunter using a smoker to harvest honey. The carvings reflect the historical significance of honey hunting in Cambodian culture. Photos by Eric Guerin.

Despite the absence of a traditional beekeeping culture in Cambodia, the practice of honey collection dates back centuries [4,6]. Historical evidence from the 12th century, such as the honey hunting scenes carved on the bas-reliefs at the Terrace of the Elephants in Angkor Thom, Siem Reap (Figure 6), confirms that wild honey collection has long been integral to Cambodian heritage. Before the introduction of modern beekeeping techniques and the exotic bee species, *A*. *mellifera*, several decade ago [3], Cambodians relied solely on the harvesting of wild honey. In contrast to its rich history of wild honey collection, the development of structured beekeeping in Cambodia has seen significant progress only in recent years [3]. Over the past ten years, Cambodia has embraced several advancements in beekeeping, including the adoption of modern hive technologies that facilitate easier management and higher yields, the initiation of extensive training programs for local beekeepers, and support from organizations such as the Maddox Jolie-Pitt Foundation. Despite these advancements, beekeeping in Cambodia still lags behind neighboring Southeast Asian countries like Thailand and Vietnam [6,32,33]. This lag can be attributed to several factors: Thailand and Vietnam have larger, more industrialized beekeeping operations that benefit from greater governmental support and more advanced beekeeping technologies. Additionally, these countries have well-established markets for honey and other bee products, supported by extensive research and development initiatives. In contrast, Cambodia’s beekeeping industry remains relatively small-scale, with limited access to modern technologies and fewer institutional supports. This discrepancy results in lower honey yields per hive, reduced profitability, and slower growth of the sector overall [6,32,33].

### 4.2. Current Status of Beekeeping and Wild Honey Collection in Cambodia

In Cambodia, honey production operates through two distinct modes: beekeeping and wild honey collection. Each method has unique characteristics and faces specific challenges.

#### 4.2.1. Beekeeping

Beekeeping in Cambodia is predominantly centered around the introduced species, *Apis mellifera*, which has become the mainstay of the country’s beekeeping industry. However, the exact number of beekeepers and hives is not well documented due to the lack of official records [6]. Surveys conducted in 2020 and 2022 estimate that there are several hundred beekeepers managing tens of thousands of hives nationwide. Currently, three crops are key to beekeeping in Cambodia: kapok (*Ceiba pentandra*), rubber (*Hevea brasiliensis*), and longan (*Dimocarpus longan*) [4,6,7]. In addition to these primary nectar sources, beekeepers also exploit several other important forage crops, including acacia, acequia palm, avocado, cassava, coconut, coffee, maize, durian, and sesame, each playing a vital role in different regions or seasons. A tentative floral calendar for some of the main cultivated Cambodian bee forage plants has been established in Table 1.

**Figure 7:**
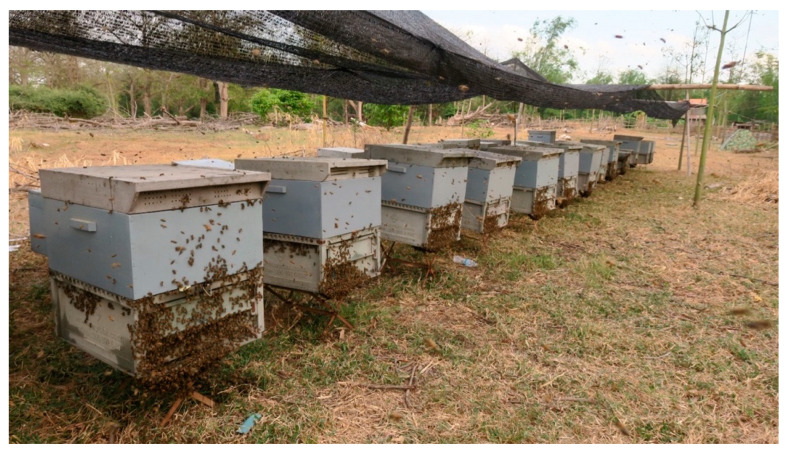
Supers (*Apis mellifera*) in a kapok tree plantation in Kampong Cham Province. Photo by Eric Guerin.

The integration of beekeeping in fruit orchards, especially longan, not only provides a source of income for beekeepers but also enhances the pollination of fruit trees, leading to increased yields and higher profits for farmers. Beekeeping has now spread widely across Cambodia, with particularly strong success in provinces rich in bee forage plants. For instance, Kampong Cham and Tbong Khmum are known for their rubber and kapok, Battambang and Pailin for longan, and Mondulkiri for rubber, albeit to a lesser extent. Most Cambodian beekeepers operate small-scale apiaries, typically maintaining between 5 and 20 hives. A minority of larger operations manage several hundred to a thousand colonies [34], often employing about two full-time workers for every one hundred hives. During peak times, such as hive migration and honey harvesting, these larger operations also rely on temporary labor to meet increased workload demands [34]. Despite the challenges, *A. mellifera* beekeeping continues to thrive in Cambodia, contributing significantly to the agricultural landscape and local economies. Migratory or transhumance beekeeping is a widely practiced strategy among Cambodian beekeepers and is used to optimize honey production and reduce reliance on artificial feeding, such as pollen and sugar syrup supplements. This practice involves relocating hives to various sites according to the flowering seasons of key crops like kapok, rubber trees, and longan. In western Cambodia, some beekeepers produce longan honey almost year-round by associating their practices with an intensive form of longan cultivation that relies on artificially induced flowering. Additionally, many beekeepers are adept at queen rearing and colony division, with some even running their own workshops for hive construction [34]. However, the market for hives and bee colonies in Cambodia is largely controlled by Vietnamese and Thai beekeepers, which significantly influences the local market dynamics. The impact of this foreign dominance on Cambodian beekeepers’ access to market opportunities warrants further investigation. A common practice in Cambodia involves harvesting honey directly from the brood box, spinning all frames that contain honey [6]. During periods of abundant honey flow, such as the kapok blooming season, supers are specifically used for comb honey production (Figure 7). Annual honey yields per hive in Cambodia vary significantly, depending on the beekeeper’s skills, hive placement, and overall apiary management. Reported yields range from as low as 6 kg to over 65 kg per hive, with the national average hovering around 30 kg. The profitability of beekeeping with *A. mellifera* in Cambodia, although not extensively studied, appears to favor those with the financial resources necessary to invest in hive care and management, potentially excluding lower-income individuals from participating fully in this sector [6].

#### 4.2.2. Wild Honey Collection

Wild honey collection is a traditional activity in Cambodia and continues to play a significant role, largely due to the attributed health benefits and medicinal properties of wild honey. Cambodian honey hunters predominantly seek out *A. dorsata* for its abundant honey yield and *A. florea* for its docile nature [6]. The amount of honey harvested from *A. dorsata* nests can vary significantly, ranging from 2 to 13 kg depending on the colony’s strength and available flower resources. Harvesting mostly occurs in the dry season, between December and April–May. With the exception of special honeys such as melaleuca honey, wet-season harvests are infrequent due to the reduced abundance of melliferous flowers and the difficulty of selling honeys with a high moisture content. In contrast, *A. florea* colony yields are typically a few hundred grams [17]. This practice supports the livelihoods of thousands, perhaps even tens of thousands, of Cambodian households with limited resources [6,35,36]. Profits from wild honey sales can be significant, accounting for up to 40 percent of total income for some honey hunting families [36]. The economic significance of wild honey collection underscores its vital role in rural Cambodian economics.

**Figure 8:**
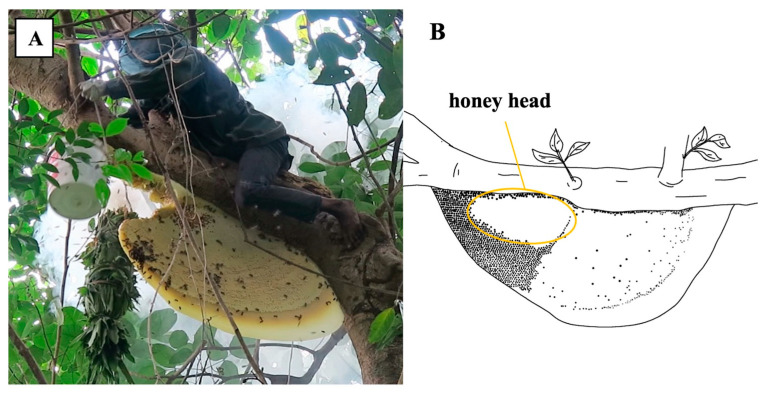
(**A**) Sustainable honey collection from *Apis dorsata* in Stung Streng Province. (**B**) Drawing of an *A. dorsata* nest. Photo by Eric Guerin.

Sustainable wild honey harvesting involves collecting only the “honey head” of the comb, which allows the colony to survive and continue producing honey, in contrast to destructive methods where the entire nest, including the brood, is removed and consumed or sold [37,38]. Such sustainable practices enable repeated harvests from *A. dorsata* nests (Figure 8) and contribute to the conservation of bee populations. However, the extraction of *A. florea* honey in Cambodia remains consistently destructive, with entire nests being removed for sale. This destructive practice undermines conservation efforts and threatens the species’ survival. Efforts to promote sustainable harvesting practices among *A. dorsata* honey hunters in the provinces of Koh Kong, Kratie, Mondulkiri, Preah Vihear, Ratanakiri, and Stung Treng [17,39] need stronger emphasis and support from environmental groups and government agencies. Sustainable collectors of *A. dorsata* honey have organized into groups and are overseen by the Cambodian Federation for Bee Conservation and Community-Based Wild Honey Enterprise (CBHE). Mondulkiri’s wild honey has been designated as a geographical indication (GI), reflecting its unique regional characteristics [36,40,41]. When practiced sustainably, wild honey collection is recognized as a non-timber forest product (NTFP), offering an alternative to logging and thus aiding in forest conservation in several Cambodian provinces [36]. It is also regarded as a mitigating factor against rural-urban migration [35]. Despite these structured efforts, numerous independent collectors across Cambodia frequently employ destructive harvesting methods, exacerbating the decline of *A. dorsata* populations [42,43]. The high demand for bee brood as a delicacy in Cambodia fuels these unsustainable practices [6], highlighting the urgent need for educational campaigns and stricter regulations.

**Figure 9:**
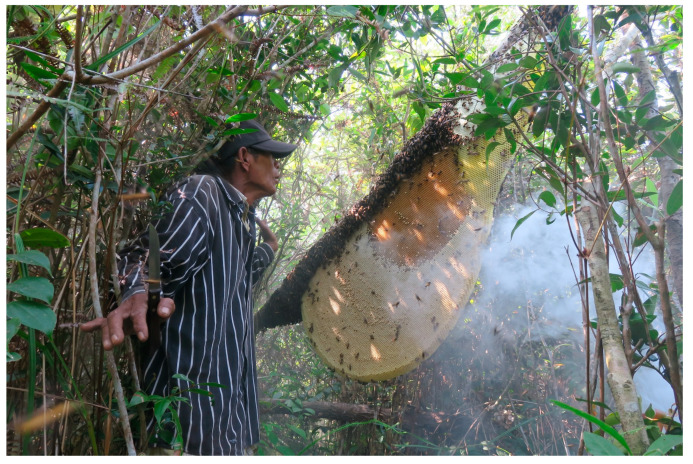
Rafter beekeeping in Siem Reap Province. As rafters are placed typically near the ground, they allow easy and safe access to the “honey head”. Photo by Eric Guerin.

Rafter beekeeping, a more sustainable approach to harvesting honey from *A. dorsata* (Figure 9), is utilized in two regions of Cambodia: the low secondary forests of the southern piedmont of Phnom Kulen in Siem Reap Province and the melaleuca (*Melaleuca leucadendron*) forests of Koh Kong Province [44]. A rafter is a simple structure, consisting of a trunk approximately 2 m long and 10 to 20 cm in diameter, supported by two vertical poles [45]. The lower surface of the rafters is coated with beeswax to encourage migrating *A. dorsata* colonies to settle on them. The rafters are generally inclined at around 30 degrees to facilitate the bees storing their honey at the upper end of the rafter, which results in a “honey head” that can be harvested sustainably without harming the brood [45,46]. As rafters are generally placed close to the ground, they allow easy and safe access for honey harvesting without the need for climbing [46,47]. Rafter beekeeping is particularly well-suited to ecosystems where natural nesting sites are scare, such as low secondary forests with branches too weak to support the weight of *A. dorsata* nests and melaleuca forests whose shaggy, papery, loose bark prevents bee colonies from nesting under the branches [44,46]. The sharp decline in local *A. dorsata* populations and extensive deforestation pose an imminent threat to the rafter beekeeping community, particularly in Siem Reap province. Protective measures for these habitats are critical to ensure the sustainability of rafter beekeeping and the preservation of local bee populations.

## 5. Honeys and Honey Value Chain

Cambodia’s bee flora can produce around ten monofloral honeys, although the production of farm honey is predominantly focused on three varieties: rubber, kapok, and longan honeys. These three contribute to approximately 90% of the national production (Table 1). Wild honey from Cambodia, commonly referred to as forest honey, presents a variety of flavors that reflect the ecosystems and seasons of their production. While typically multifloral, certain Cambodian forest honeys exhibit a dominant nectar source. The unique flavors of these honeys offer potential for niche marketing and specialty product development. The quality and traceability of sustainably collected wild honey have notably improved, largely due to the organized efforts of NGOs such as the World-Wide Fund for Nature and the Non-Timber Forest Products Exchange Program (NTFP-EP) [48]. Efforts by the CBHE and the GI Mondulkiri Wild Honey initiative are underway to implement a participatory guaranteed system. This system ensures that honeys meet sustainability, quality, and traceability standards, which are critical for maintaining consumer trust and expanding market access.

In Cambodia, honey is often prized more for its medicinal or cosmetic uses, such as skincare, rather than as a food item. Wild honeys, particularly those from *A. dorsata*, command a higher value than farm-produced honeys, which are often perceived by consumers as less medicinal and more susceptible to adulteration. This perception underscores the importance of consumer education on honey qualities. Awareness of distinction, such as between raw and pasteurized honey, is limited. Crystallized honey is commonly mistaken for adulteration, and consequently, it is avoided by consumers who associate crystallization with added sugar. Educational campaigns could help correct these misconceptions, potentially increasing market demand for crystallized honey. Other bee products like bee pollen, royal jelly, and propolis are not widely recognized by Cambodian customers. Introducing these products through educational and marketing efforts could tap into new customer segments. The market for bee products in Cambodia has transitioned from a focus on home consumption and traditional medicine to a broader commercial sphere with various stakeholders. Honey is sometimes sold directly to consumers by collectors and beekeepers, though the bulk is typically traded to wholesalers or retailers. Large retailers prioritize price and delivery consistency when selecting suppliers, while authenticity is the chief concern of honey traders. The development of quality control facilities in Cambodia remains crucial for improving product standards and consumer confidence. Small-scale retailers, including convenience stores, souvenir shops, cafes, beekeepers’ shops, delicatessens, hotels, and online shops, tend to offer Cambodian honeys. Conversely, supermarkets are more likely to stock imported varieties [15]. Although women are underrepresented in honey production, they play a significant role in the honey trade [6,17].

**Figure 10:**
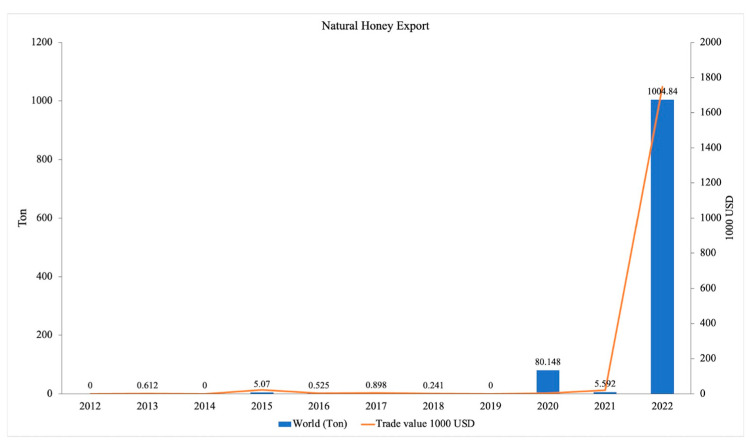
Trend of natural honey exports from Cambodia over time from 2012 to 2022. Data from World Integrated Trade Solution (WITS, 2023) reflect the export volume of natural honey, primarily produced by *Apis mellifera*.

Cambodia exports natural honey to other countries or regions such as Korea, Hong Kong (China), United States, Singapore, Switzerland, Indonesia, and Japan [49]. The report on natural honey exports from Cambodia covers the period from 2012 to 2022 (Figure 10). According to data from the World Integrated Trade Solution, honey exports from Cambodia have been insignificant until recently. The reasons for the remarkable increase in exports in 2022 remain unexplained but could be an indirect consequence of antidumping taxes imposed by the US Government on honey imports from several countries, including Vietnam. Further research could elucidate the specific drivers behind this recent surge in exports, providing valuable insights for future trade strategies.

## 6. Pests and Diseases

While exploring the vibrant dynamics of Cambodia’s beekeeping industry, it is essential to consider the health of bee populations, as they face numerous threats from pests and diseases. Globally, and particularly in Southeast Asia, significant research efforts are directed towards understanding and mitigating these threats. However, Cambodia’s capacity to study and manage bee health issues lags behind other countries, potentially impacting the sustainability and productivity of its beekeeping practices.

Compared to a broad range of countries, including Southeast Asia, where the study of bee diseases and parasites is well-established [9], research in Cambodian apiaries remains relatively limited [34]. This gap in knowledge and management capability is critical, as bee health is integral to the viability of beekeeping industries worldwide. The underdevelopment in diagnosing bee diseases and pests in Cambodia can be attributed to both technical deficiencies and resource constraints. There is a need for more robust training programs, better access to diagnostic tools, and increased funding for research. Cambodian beekeepers have observed ectoparasitic mites such as *Varroa* and *Tropilaelaps*, and diseases like chalkbrood in their apiaries. To manage mite infestation, they report using acaricides such as Amitraz in strip or spray forms, as well as formic acid [34]. Additionally, sacbrood disease has been noted in *A. cerana* colonies. Investing in education on integrated pest management could enhance the effectiveness and safety of these treatments. Other diseases, such as European foulbrood and nosema, have not been reported by Cambodian beekeepers but are likely present due to the extensive importation of bee colonies, particularly from Vietnam and Thailand, where these diseases are prevalent [32,33]. Implementing stricter import controls and regular health assessments for imported colonies could help prevent the introduction and spread of these diseases.

**Figure 11:**
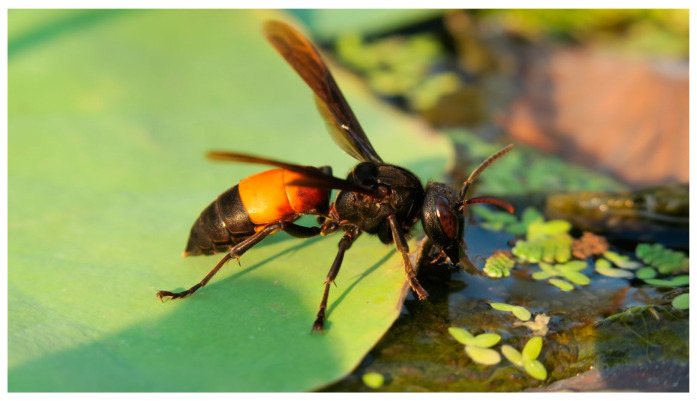
Lesser banded hornet, *Vespa affinis*: one of the natural predators of honeybees in Cambodia. Photo by Eric Guerin.

Cambodian apiaries also face predation from several predators, notably hornets, bee-eaters, and ants. Four species of hornet (*Vespa affinis* (Figure 11), *Vespa soror*, *Vespa tropica*, and *Vespa velutina*) and five species of bee-eaters (*Merops leschenaultia*, *Merops orientalis*, *Merops philippinus*, *Merops viridis*, and *Nyctyornis athertoni*) have been identified [32,33]. Beekeepers often use nets to protect their hives during periods of high predation by bee-eaters. Further research into alternative protective measures could provide beekeepers with additional strategies to safeguard their colonies.

## 7. Challenges and Policy Recommendations for the Conservation of Cambodia’s Native Bees and the Development of Honey Production

### 7.1. Challenges for the Conservation of Cambodia’s Native Honeybees

Insufficient scientific documentation on the biology and population trends of Cambodia’s native honeybees, combined with limited recognition by government entities of their socio-economic and environmental importance, significantly hampers the development of conservation policies [50]. The rapid destruction of natural habitats, particularly forests, due to economic development, deprives native bees of essential nesting sites and floral resources [51,52,53]. Agricultural intensification contributes further to habitat loss, a reduction in bee flora diversity, and increases bee exposure to pesticides [54,55,56,57,58]. These factors collectively undermine the survival and health of bee populations. Furthermore, Cambodian farmers’ limited understanding of bee’s role in crops pollination often leads to the mistaken treatment of bees as pests, exacerbating pesticide issues [34]. The importation of exotic stingless bees for melipoculture and subspecies of *A. cerana* poses another risk, potentially leading to ecological issues if these species become invasive or if their genetic material introgresses with wild populations [59,60,61]. This scenario demands careful management and strict regulation to prevent genetic interference.

### 7.2. Challenges for the Development of Honey Production in Cambodia

The absence of official honey production data and a lack of documentation on bee floral resources impedes efforts to enhance the beekeeping sector. Restricted access to productive apiaries, particularly in longan orchards and kapok plantations, coupled with the indiscriminate use of pesticides and widespread misunderstanding about pollination, severely limits potential honey yields and industry growth. Strategic interventions are crucial to improve access to these essential resources. The future of wild honey production is at risk due to diminishing forest areas and the decline of native bee populations. Inappropriate pesticide practices, such as excessive spraying, the use of highly toxic chemicals, and the mixing of different pesticides, not only limit the availability of suitable apiaries but also escalate the risk of colony losses due to pesticide poisoning [34,51,62,63,64]. The limited area of kapok plantations, compared to other key floral resources like longan and rubber trees, restricts the number of hives available for transhumance beekeeping. The environmental impact of this practice is concerning, particularly because of the significant carbon footprint associated with transporting hives and the potential for spreading bee parasites and diseases [65].

Cambodian beekeepers’ limited knowledge of bee health, diseases, and parasites could undermine the productivity of their apiaries and expose the industry to new pathogens [65]. The importation of bee colonies and potentially queen bees from abroad brings the risk of introducing new diseases or parasites, which could spread more easily due to common practice of migratory beekeeping [33,66]. Strengthening local expertise and capabilities in bee health management is therefore crucial. The beekeeping sector’s growth is hindered by a shortage of skilled personnel and difficulties in accessing financial resources, which restricts the expansion of beekeeping and wild honey enterprises, particularly for low-income families [34,36,67]. Moreover, the inadequate organization and representation of Cambodian beekeepers impede their advocacy efforts to integrate beekeeping with agricultural development and affect their ability to market their products effectively. Developing stronger beekeeper associations could bridge this gap.

In the long term, the honey production sector may face challenges from climate change, such as the loss of floral resources, reduced colony foraging capacity, the emergence of new bee pathogens, and the rapid decline of *A. dorsata* populations, a primary source of Cambodia’s wild honey [68,69,70,71,72]. Additionally, Cambodian honey marketing struggles with two main issues: customers’ lack of knowledge and trust in local bee products, perpetuated by misconceptions about honey and weak commercial relationships between beekeepers and major retailers. Moreover, the higher cost of Cambodian honey compared to that from neighboring countries puts it at a competitive disadvantage. Addressing these marketing challenges through consumer education and strengthening supply chain relationships is vital to enhancing the sector’s competitiveness.

## 8. Future Perspectives

### 8.1. Conservation Strategies for Cambodia’s Native Honeybees: Integrating Ecological, Socio Economic, and Regulatory Measures

As we have mentioned in our introduction, the four native honeybee species in Cambodia are not currently at risk of extinction, but their significant socio-economic and ecological roles, such as pollinating ecosystems and crops, producing honey, and supporting the livelihoods of low-income households, warrants dedicated conservation efforts. Threats such as habitat loss, agriculture and beekeeping intensification, unsustainable honey hunting, and climate change underline the urgent need for well-defined conservation policies. Integrating bee conservation strategies with broader conservation goals, including deforestation reduction and the promotion of sustainable agriculture, is essential. Educational initiatives targeting both farmers and the general public should be implemented to raise awareness about the ecological benefits of bees and their essential role in agriculture. Stricter regulations on pesticide use should be developed and enforced and farmers trained in integrated pest management practices. Additionally, specific regulations are also crucial, particularly to promote sustainable wild honey collection and to counteract the adverse effects of beekeeping intensification. Enhancing the documentation of the biology, status, and trends of Cambodia’s native bee populations is a pivotal first step in informing policymakers and shaping effective mitigation strategies [5]. Conservation efforts must encompass all native bees, not merely honey-producing species. Thus, completing the inventory of all bee species in Cambodia is vital, as many species potentially new to science remain undocumented [5].

Interdisciplinary collaborations that engage both scientists and local communities through participatory research programs are instrumental in documenting wild bee species. These collaborations not only enhance data collection but also provide significant educational benefits, thereby contributing to broader conservation goals. The rich knowledge held by wild honey collectors about native bee flora and biology is invaluable for developing effective bee and ecosystem conservation strategies and should be preserved. The conservation and restoration of bee habitats, particularly forests, must take into account the migratory routes of bees. Implementing bee-friendly agricultural practices, such as agroecology and agroforestry, benefits Cambodian smallholder farmers by improving crop pollination and overall ecosystem health [73,74,75,76]. It is essential to encourage wild honey collectors to adopt non-destructive harvesting methods that spare the bee brood, thus allowing for sustainable multiple harvests. Educating consumers on the harmful impact of consuming bee brood is vital for protecting native bee populations. Bee conservation policies should also address the potential negative impacts of conventional beekeeping on native bees. Regulating the importation of live bees is crucial to prevent the introduction of foreign parasites and diseases to native bee populations. In natural and protected areas, beekeeping should be regulated to avoid competition between *A. mellifera* bees and wild native bees [40]. Furthermore, the development of beekeeping with native species, such as *Apis cerana* or stingless bees (*Tetragonula pagdeni*), should be conducted exclusively with Cambodia’s local subspecies. This approach is essential to prevent genetic introgression and preserve the integrity of local bee populations. The importation of exotic stingless bee species must be prohibited to safeguard Cambodia’s ecosystems from the risks posted by invasive species [77,78].

Several initiatives for the conservation of wild bees have been undertaken in Cambodia. Notably, UNESCO launched a national campaign in 2022 to raise awareness about the impact of consuming bee brood on *Apis dorsata* populations. Additionally, programs promoting agroecology and agroforestry, such as the GRET-APICI project in Siem Reap province, help reduce pesticide use and preserve bee habitats [79]. Moreover, NTFP-EP has introduced programs in the provinces of Koh Kong, Kratie, Mondulkiri, Preah Vihear, Ratanakiri, and Stung Treng to promote sustainable harvesting methods for *Apis dorsata* honey [39]. However, to date, these initiatives remain too isolated and limited in scale to significantly impact the decline in wild bee populations. Reversing the current trend will require an ambitious policy to protect pollinators in general and wild bees in particular. UNESCO’s roadmap for developing a national plan for sustainable beekeeping and native bee conservation in Cambodia marks an important first step [39]. Effective pollinator protection will require integrating the roadmap’s recommendations—and eventually the national plan—into the strategic plans of key ministries, including those of agriculture, forestry, and the environment.

### 8.2. Enhancing Beekeeping in Cambodia: Strategies for Conservation, Expansion and Sustainability

To safeguard productive apiaries and promote their expansion, comprehensive documentation of both cultivated and wild bee floral resources in Cambodia is essential. Special priority should be given to preserving and expanding kapok plantations, which are vital for the survival and development of beekeeping industry. Exploring the viability of sedentary beekeeping models, which could provide a financially sustainable alternative to migratory practices, should also be investigated. Educational campaigns need to raise farmers’ awareness of the differences between agricultural pests and pollinators, highlighting the beneficial effects of the latter on crop yields. Collaborative applied research programs involving researchers, beekeepers, and farmers can help identify pesticide alternatives, fostering a mutually beneficial relationship. A suite of technical, legal, and educational measures regarding pesticides is required to reduce managed bee colony mortality from pesticide poisoning, improve beekeepers’ access to floral resources, and minimize bee product contamination from pesticides [65]. This should include adopting biological and agroecological pest control methods and establishing best practices for pesticide application. Legal steps might involve restricting particularly bee-toxic pesticides during registration and mandating pollinator risk and protection guidelines on labels [65].

Educational efforts should focus on training pesticide users about the importance of pollinators, the detrimental effects of pesticides on bee populations, and proper pesticides usage [80,81]. Conserving and restoring habitats, particularly forests critical to native honeybee like *A. dorsata*, is essential for maintaining Cambodia’s wild honey production. The establishment of beekeeping research and training centers will not only enable Cambodia to engage in regional bee-related research programs but also facilitate the dissemination of beekeeping knowledge and practices among local beekeepers. Implementing Good Apiculture Practices provides beekeepers with a reference for their daily activities and enhances the quality of bee products.

Policies restricting bee imports must be bolstered to prevent the entry of exotic bee diseases and pests, genetic contamination of native bees, and the introduction of invasive species. These measures should be implemented by educational initiatives focusing on critical beekeeping practices, such as queen rearing, swam control, artificial swarming, and selective breeding. Registration and inspection of managed bee colonies along with monitoring and documentation of hive movements are also recommended [65]. Given that meliponiculture requires less skills and investment than honey beekeeping, its promotion can engage economically disadvantaged households in beekeeping and potentially enhance pollination on small-scale farms [69,82]. Forming honey producers’ associations can consolidate resources to negotiate better access to apiaries with farmers and landowners, address pesticides, bee flora, and beekeeping concerns with government entities, refine beekeeping and honey collection methods, and market Cambodian bee products domestically and internationally. To boost the domestic honey trade, Cambodia should implement national honey standards in line with Codex Alimentarius, as practiced in other regional countries [83,84], and enhance quality and authenticity testing facilities. Building capacity among honey value chain stakeholders in quality, authenticity, processing, packaging, and marketing of honey is also necessary. Moreover, the development of honey dehumidification systems that maintain product quality and processing methods for honeys with high crystallization tendency, like rubber honey, are essential for the commercial viability of currently undervalued honeys. Strengthening the connections between honey producers and major retailers, such as supermarkets and duty-free shops, can broaden market access and meet the growing consumer demand for local products [34].

## 9. Conclusions

Conservation of native honeybees and the advancement of beekeeping in Cambodia face significant challenges, including habitat loss, harmful agricultural practices, wild bee brood consumption, and limited local expertise. The introduction of non-native species further threatens indigenous bee populations. To effectively address these issues, an integrated approach is necessary, combining policy reform, community engagement, and technological advancement with the promotion of sustainable practices. By focusing on these strategies, Cambodia can improve the sustainability of its bee populations and the economic viability of its beekeeping industry, thereby supporting both ecological health and agricultural productivity. Such integrated measures are essential for sustaining bee populations and supporting the economic benefits they provide. They ensure that conservation and beekeeping efforts are aligned with broader ecological and agricultural policies, enhancing overall effectiveness.

## Figures and Tables

**Table 1:** Flowering calendar of the plant species recovered in the major of honey in Cambodia.

Common Name	Scientific Name	Foraging Activity				Flowering Period (Month of Production)										Percentage of Honey Production
		Nectar	Nectar + Pollen	1	2	3	4	5	6	7	8	9	10	11	12	
Kapok	*Ceiba pentandra*		✓													35
Longan (natural bloom)	*Dimocarpus longan*		✓													15
Longan (triggered bloom)	*Dimocarpus longan*		✓													
Rubber	*Hevea brasiliensis*	✓														40
Acacia	*Accacia mangium*	✓														Unknown
Coffee	*Coffea canephora*		✓													Unknown
Sesame	*Sesamum indicum*		✓													Unknown

Note: Columns or rows shaded in gray indicate the blooming period of plant species. ✓ indicates presence.

## Data Availability

Not applicable.
